# Fetal inflammatory signals regulate maternal investment during marsupial pregnancy

**DOI:** 10.1371/journal.pbio.3003670

**Published:** 2026-02-24

**Authors:** Daniel J. Stadtmauer, Jamie D. Maziarz, Oliver W. Griffith, Gunter P. Wagner

**Affiliations:** 1 Department of Ecology & Evolutionary Biology, Yale University, New Haven, Connecticut, United States of America; 2 Department of Evolutionary Biology, University of Vienna, Vienna, Austria; 3 Hagler Institute of Advanced Studies, Texas A&M University, College Station, Texas, United States of America; National Institute of Environmental Health Sciences, UNITED STATES OF AMERICA

## Abstract

Marsupial pregnancy is strikingly short: placental attachment in the gray short-tailed opossum *Monodelphis domestica* lasts only two days. The attachment period is characterized by a spike in inflammatory signaling, development of an expanded uterine capillary network, and exponential fetal growth. This brevity has historically been attributed to a maternal immune response to fetal contact that only eutherian mammals have evolved mechanisms to tolerate. However, several inflammatory cytokines, including interleukin-1A (IL-1A) and interleukin-6 (IL-6), are produced primarily by fetal cells. We hypothesized that placental cytokines function as solicitation signals that increase maternal investment. To test this, we treated pregnant opossums with inhibitors of IL-1 and IL-6 during the rapid growth phase. Inhibition of IL-1 and IL-6 signaling significantly increased average biomass per fetus (+14% and +12%), and as such these signals impose costs, rather than direct benefits, to intrauterine growth. However, controls showed greater surviving litter sizes than IL-1-inhibited animals, suggesting that IL-1A promotes offspring survival. Single-cell transcriptomes reveal that maternal vascular endothelial cells, perivascular cells, and fibroblasts are the primary targets of fetal IL-1A, and that maternal cells simultaneously up-regulate IL-1 antagonists *IL1R2* and *IL1RN* late in gestation, suggesting maternal resistance to fetal signaling. Placental transcriptomics reveals that the cytokine surge is restricted to the final day of pregnancy when placental cells fuse to form syncytial knots, and that these cells produce additional vasomodulatory signals including a truncated isoform of *VEGFA*. We propose that marsupial fetuses co-opted inflammatory signaling to perform a novel solicitation function promoting their and their littermates’ survival, possibly by altering maternal vascular development.

## Abstract 2

Marsupial pregnancy is short and characterized by a spike in inflammation which has been proposed to represent a maternal immune response to fetal contact that limits gestation. This study shows that in short-tail opossums, inflammatory signaling shape maternal investment vascular development during pregnancy to promote offspring survival.

## Introduction

The life history dichotomy between eutherian (placental) mammals and marsupials has long puzzled biologists. Marsupials give birth to highly altricial neonates, which must complete development attached to the nipples, whereas eutherian mammals have evolved extended gestation that can produce precocial offspring. A central question is whether marsupial short gestation reflects an intrinsic constraint or an alternative adaptive strategy. It has been proposed that marsupials are developmentally constrained from evolving extended gestation due to a maternal immune response to paternally-derived fetal antigens that only eutherian mammals have evolved mechanisms to tolerate [1]. Major macroevolutionary trends, including the extinction of many South American marsupials in the late Cenozoic upon introduction of eutherians to the continent, have been attributed to these differences in reproductive biology [1,2]. Alternatively, it has been proposed that marsupials have short placentation due to selection for early birth and highly-specialized lactation, which transfers similar amounts of biomass postnatally, making abrupt parturition a reproductive strategy rather than a closed developmental opportunity [3–5]. These hypotheses make different predictions about whether late-gestation inflammation is a constraint or a functional component of fetal development. The immunological constraint hypothesis predicts maternal, antigen-driven, runaway rejection-like inflammation at term, whereas the lactation hypothesis predicts that endogenous signaling mechanisms elicit parturition.

Bulk transcriptomics has lent apparent support to the immunological constraint hypothesis by revealing acute production of pro-inflammatory cytokines at the utero-placental interface during the final 1–2 days of pregnancy in the gray short-tailed opossum [6,7] and the fat-tailed dunnart [8]. However, two elements not predicted by the original immunological constraint model have also been discovered. First, the response is predominantly driven by innate immunity—inflammation and granulocyte recruitment—rather than adaptive immunity, which mediates graft rejection [6,9]. Second, many cytokines have been discovered to be produced by the fetus via placental trophoblast cells, contrary to an antigen- or damage-induced inflammatory response, which should be maternally driven [[10,11],summarized in [12]]. Together, these findings suggest that the inflammatory program may be regulated and fetal-driven, rather than a maternal rejection response.

Inflammation is not merely destructive: beyond defense against non-self invaders, it induces local edema, angiogenesis, and tissue remodeling [13]. These sequelae hold a latent potential for evolutionary co-option to facilitate nutrient delivery. This potential has been realized in multiple contexts, including the origin of mammalian lactation, where NF-κB and antibacterial enzymes have acquired secondary roles regulating resource allocation [14], as well as an evolutionarily novel brooding tissue in ricefish whose development depends upon inflammatory processes [15]. Previously, we speculated that marsupials may have leveraged these vasomodulatory effects to enhance nutrient provisioning during placentation [12]. The repeated co-option of inflammation in evolutionarily novel modes of offspring provisioning raises the question: *does inflammation in marsupial pregnancy benefit the fetus*?

**Fig 1:**
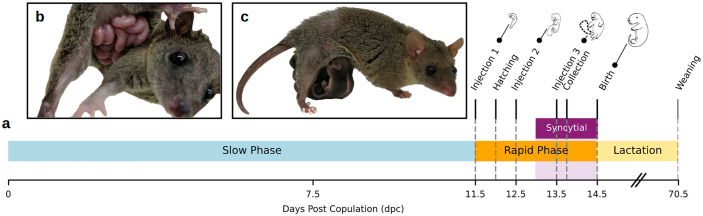
Pregnancy timeline in the opossum. **(a)** Timeline of opossum development showing the slow vs. rapid developmental phases and the onset of syncytial knot formation. Shell coat hatching occurs at 12 dpc and syncytialization on day 13. Lactation from 14.5 to 70.5 dpc is linearly compressed for clarity. Timing of anti-inflammatory injection experiments are marked. **(b)** *M. domestica* litter on the day of birth. **(c)** Litter ~1 month into the lactation period. Embryo illustrations in (a) are drawn after [66] whose copyright term has expired to enter the U.S. public domain. Photos in (b) and (c) are copyright of O. Griffith and D. Stadtmauer, respectively.

In the gray short-tailed opossum *Monodelphis domestica*, fetuses degrade the shell coat approximately 12 days postcopulation (dpc), and receive oxygen and nutrients from uterine vasculature and glands through the placenta (Fig 1) [16]. Trophoblast cells fuse on day 13 to form multinuclear aggregates known as syncytial knots [17]. These cells produce *IL1A*, *IL6*, and other cytokines, and their development coincides with rapid growth of the fetus and glandular opening and the development of a subepithelial capillary network in the endometrium [11,18–20]. This brief placentation, which has also been called the “exponential rapid growth phase” [20], is when the majority of intrauterine embryonic development occurs (Fig 1). Here, we test whether fetal-driven inflammation directly influences maternal investment by identifying the cellular targets of placental inflammatory cytokines and pharmacologically inhibiting them to identify their functional consequences. We identify potential inflammatory solicitation signals by comparing placental ligand expression between day 13.5 and day 12.5 to isolate changes associated with syncytial knot formation. We analyze single-cell transcriptomes from the 13.5 dpc interface to identify potential maternal target cells of fetal signals. Lastly, we tested the effects of inhibiting three fetal cytokine pathways – interleukin-1A (IL-1A), interleukin-16 (IL-6), and PGE_2_ – on litter size and fetal biomass to gain insights into the functional significance of these signals for maternal investment. Our findings reveal that inflammatory signals play surprisingly complex roles in shaping maternal investment beyond those predicted by the immunological constraint hypothesis, and suggest that cytokines have acquired novel reproductive functions in marsupial pregnancy.

## Results

### Placental cytokine production coincides with syncytial knot development and targets maternal vasculature and glands

**Fig 2:**
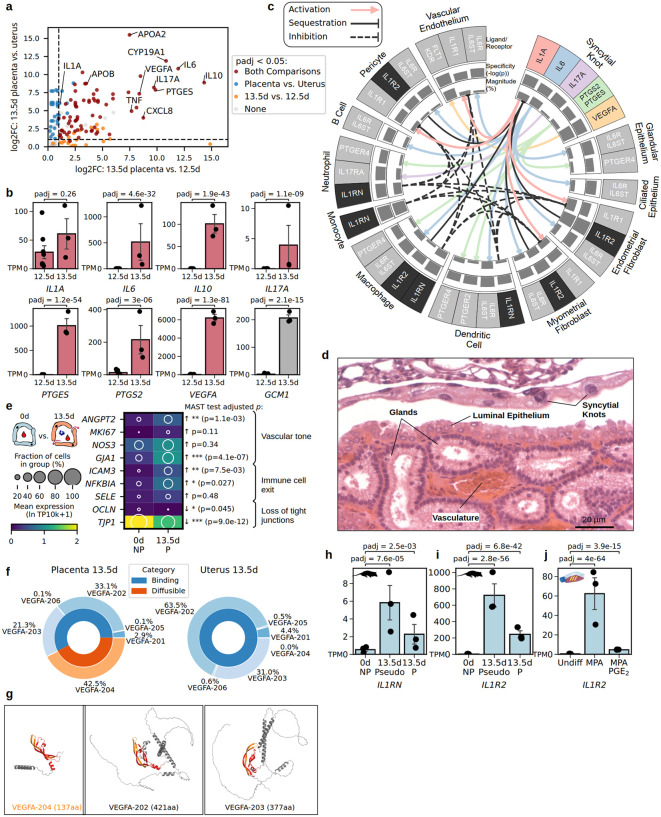
Temporal and cell type specificity of inflammatory ligands, receptors, and physiological inhibitors in the last day of opossum pregnancy. **(a)** Log2-transformed fold change in ligand gene expression (log2FC) between 13.5-day placental membranes (*n* = 3, this study) and 12.5-day placental membranes (*n* = 8, [21]) on the horizontal axis, and comparison between 13.5-day placental membranes and 13.5-day-equivalent pseudo-pregnant uterus (*n* = 3, [18]) on the vertical axis. Points are colored by pyDESeq2 Wald test signifiance. Full statistics are in S1 Data. **(b)** Total expression and differential expression statistics of select genes before (12.5d) and after (13.5d) syncytialization from the same data sets as (a). **(c)** Inferred cell-cell ligand-receptor interactions of select inflammatory cytokines originating from syncytial knots and their inhibitors at the 13.5 dpc utero-placental interface (*n* = 2, [11]). Magnitude barplots reflect the percent of cells in the cell type cluster with nonzero expression of the ligand or receptor gene (or lesser of the two for pairs like PTGS2 + PTGES). Specificity barplots reflect LIANA+ robust rank aggregate p-value scores. Only interactions exceeding expression levels of 40 TPM and 15% of cells in the cluster for ligand and receptor are plotted. Full statistics are in S2 Data. **(d)** Hematoxlin and eosin-stained 13.5 dpc uterus showing apposition of syncytial knots to uterine lumen and underlying vasculature. **(e)** Expression of markers of vascular permeability, proliferation, leukocyte migration, cytokine response, and tight junction integrity in aggregated vascular endothelial cell transcriptomes from non-pregnant [23] and 13.5 dpc [11] uteri. P-values represent Benjamini-Hochberg corrected *p*-values from MAST [61]. Values are in S3 Data. **(f)** Relative *VEGFA* isoform abundances in 13.5 dpc placenta (left) vs. 13.5 dpc pregnant uterus (right). Inner rings show proportions of transcripts encoding Binding (orange) and Diffusible (orange) peptides. Fetal-specific expression of the truncated splice variant *VEGFA-204*, equivalent to human *VEGF*_*111*_. Data points are in S4 Data. **(g)** Predicted protein structures [67] of *VEGFA-204* compared to co-expressed *VEGFA-202* and *VEGFA-203* show conservation the receptor-binding domain encoded by exons 2 and 3 (red and orange) but differ in the presence or absence of additional heparin and neuropilin-binding domains encoded by exons 5–7. **(h, i)** Uterine expression of *IL1RN* (h) and *IL1R2* (i) in bulk transcriptomes from non-pregnant (*n* = 3, “0d NP”), 13.5 dpc-equivaelent pseudo-pregnant (*n* = 3, “13.5d Pseudo”), and 13.5 dpc pregnant (*n* = 3, “13.5d P”) individuals [18]. **(j)** Expression of *IL1R2* in in vitro cultured opossum endometrial stromal fibroblasts in response to 2 days treatment with either 1 µM of the progesterone analog medroxyprogesterone 17-acetate (MPA), 1 µM MPA plus 10 µM PGE_2_ (MPA/PGE2) or growth medium-only control (Undiff) from (*n* = 3/treatment, [27]). *P*-values in (b) and (h–j) are Benjamini–Hochberg-corrected Wald test results from pyDESeq2 on read counts (padj), whereas *p*-values in (e) are likelihood ratio test *p*-values from MAST.

First, we sought to identify the fetal signaling genes that turn on when syncytial knots develop on day 13 of pregnancy. We captured placenta-specific gene expression by laser microdissection of 13.5 dpc extraembryonic membranes to exclude maternal tissue, and compared the resulting micro-bulk transcriptomes to published transcriptomes of the 12.5 dpc extraembryonic membranes [21], as well as to uterine transcriptomes from pseudo-pregnant females, i.e., females exposed to male pheromones to induce pregnancy-like endometrial remodeling [18], to represent endogenous maternal signaling. We subset the resulting transcriptomes to genes encoding secreted ligands or enzymes catalyzing their synthesis using a gene list from a previous study [11], and conducted differential gene expression testing (Fig 2a and 2b and S1 Data). Genes showing both temporal and fetal-specific enrichment included the cytokines *IL6*, *IL10*, *IL17A*, and *CXCL8*, as well as *PTGES*, which catalyzes production of the inflammatory mediator prostaglandin E_2_ (Fig 2a). The vascular endothelial growth factor *VEGFA* also showed pronounced increases, reaching >6,000 transcripts per million (TPM) in the day 13.5 whole placenta, a more than 300-fold increase from day 12.5 (Fig 2b). To investigate whether these changes correspond with syncytial trophoblast cell differentiation between days 12.5 and 13.5, we compared the expression of *GCM1*, a transcription factor previously shown to be specific to syncytial knot cells [11], and found that its expression increased around 100-fold at 13.5 dpc (Fig 2b). Together, these patterns suggest that inflammatory cytokine production begins after 12.5 dpc, coinciding with both the shedding of shell coverings and the development of syncytial knots.

We next asked which maternal cell types are competent to respond to fetal cytokines. Using single-cell transcriptomes from the 13.5 dpc pregnant uterus [11], we inferred ligand-receptor communication from fetal syncytial knot cells to maternal cell types using LIANA+ [22] (S2 Data). This analysis identified potential signaling between fetal IL-1A and maternal vascular endothelial cells, pericytes, and endometrial stromal fibroblasts, which all expressed the receptor *IL1R1*, while this receptor was largely unexpressed in maternal immune cells (Fig 2c). The IL-6 receptor *IL6R* and its co-receptor *IL6ST* were expressed in both immune (macrophages, dendritic cells, and B cells) and non-immune (ciliated glandular epithelial cells) cells, suggesting widespread signaling (Fig 2c). PTGER4, a receptor for PGE_2_, was expressed in maternal immune cells and glandular epithelium (Fig 2c). The fact that non-immune cells also expressed receptors for placental cytokines, including cells of the vasculature and glands that could plausibly regulate nutrient allocation, suggested that they may have non-immune roles. Indeed, the placenta is separated from maternal endothelial cells and their associated pericytes by only a thin uterine epithelium (Fig 2d), making paracrine diffusion a plausible route by which fetal ligands could act on the maternal vasculature and other adjacent targets.

### Endothelial cell gene expression changes at the time of fetal contact, while syncytial knots produce a truncated *VEGFA* isoform

If fetal signals influence maternal allocation, the most direct routes would be through the vasculature and glands. To investigate possible effects of fetal cytokine signaling on the vasculature, we compared expression of known permeability markers between aggregated endothelial cell transcriptomes from the 13.5 dpc [11] and non-pregnant uterus [23] (S3 Data). Maternal endothelial cells at 13.5 dpc expressed higher levels of vasodilation markers *ANGPT2* and *NOS3* [24,25], proliferation marker *MKI67*, permeability marker *GJA1*, and leukocyte adhesion molecules (*ICAM3*, *SELE*), whereas tight junction markers *OCLN* and *TJP1* were downregulated compared to the non-pregnant state (Fig 2e). These changes provide transcriptomic evidence that the maternal endothelium is not in a homeostatic state at 13.5 dpc, but instead exhibits a signature consistent with dynamic vascular function during the narrow placentation window.

Fetal cells also expressed factors consistent with instructive control of maternal vascular development. *VEGFA* expression was highest in syncytial knot cells (>7,000 TPM) (Fig 2c and S2 Data), and isoform-level quantification (S4 Data) showed that 42.5% of placental *VEGFA* transcripts lacked exons 5–7 (Fig 2f), encoding a truncated peptide (VEGFA-204) analogous to human VEGF_111_, which is more distantly diffusible and potently angiogenic due to the absence of heparin or neuropilin binding sites and protease cleavage motifs (Fig 2g) [26]. This isoform was not expressed in maternal tissues, which only transcribe the poorly-diffusible long *VEGFA* isoforms used in normal tissue homeostasis. Together with endothelial transcriptomic changes, this finding supports a model in which fetal cells are functionally specialized to modulate maternal vascular development late in gestation.

### Maternal cells express IL-1 antagonists *IL1RN* and *IL1R2*

We next asked whether maternal tissues deploy counter-regulatory mechanisms that could constrain cytokine signaling during late gestation. Maternal immune cells were found to express *IL1RN*, a competitive IL-1 receptor inhibitor (Fig 2c), and, several maternal cell types expressed high levels of *IL1R2*, a decoy receptor that sequesters IL-1A, in particular endometrial fibroblasts, which expressed this gene in excess of 9,000 TPM (Fig 2c). Comparison of published whole-uterine transcriptomes [6] revealed significant upregulation of *IL1RN* (>5-fold) and *IL1R2* (>100-fold) in 13.5 dpc pregnant relative to non-pregnant uteri (Fig 2h and 2i). To probe whether expression of these inhibitors resulted from a negative feedback response to fetal *IL1A* or an autonomous maternal response, we compared 13.5 dpc uterine transcriptomes to pseudo-pregnant transcriptomes [18]. Pseudo-pregnant uteri showed similarly elevated levels of *IL1RN* and *IL1R2* (Fig 2h and 2i), suggesting that maternal IL-1 antagonism is under maternal hormonal control. Published transcriptomes from in vitro cultured opossum endometrial stromal fibroblasts [27] reveal that *IL1R2* expression in this cell type is significantly upregulated after exposure to exogenous progestin (Fig 2j), suggesting that maternal progesterone, which peaks 11−12 days after ovulation [28], coordinates timing of the IL-1 antagonism response. These results suggest that maternal cells have evolved dynamic counter-regulation of fetal IL-1A, and that the peak of IL-1 inhibition is restricted to the late stage of pregnancy by maternal hormones.

Our combined findings that fetal cytokines have complementary receptors expressed in both immune and non-immune maternal cell types, including those such as vascular and glandular cells that could plausibly regulate nutrient allocation, and that the mother dynamically downregulates part of this signaling, is consistent with what would be expected if placental cytokines function as solicitation signals. We therefore hypothesized that fetal signals function to increase maternal nutrient transfer, and sought to test this prediction by perturbing these signals and quantifying the effects on fetal growth and survival.

### IL-1 inhibition leads to fewer, larger surviving offspring

**Fig 3:**
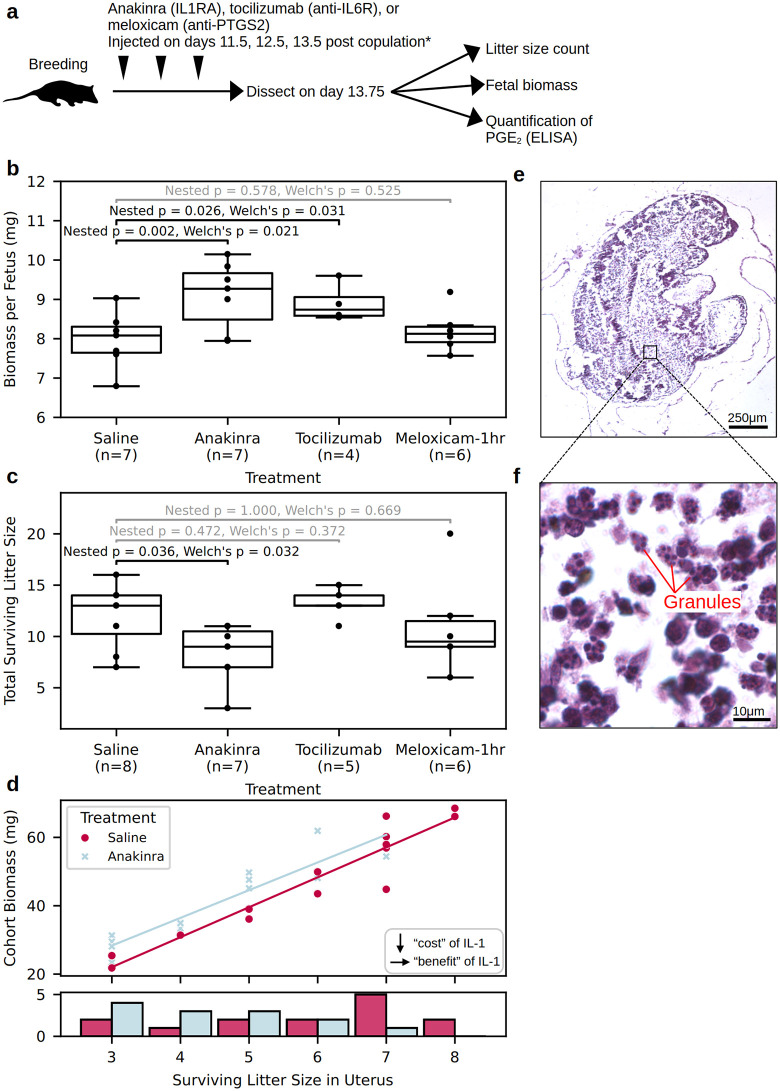
Anti-inflammatory intervention into opossum pregnancy. **(a)** Experimental design. **(b, c)** Effects of anakinra, tocilizumab, and meloxicam on fetal biomass (b) and litter size (c). The middle lines mark the median, boxes mark the interquartile range (IQR), and whiskers extend to the furthest points not exceeding 1.5× the IQR. Full measurements are in S5 Data. **(d)** Hematoxylin and eosin histology of resorbed embryo remnants. **(e)** Zoomed panel from (d) showing resorbed matter cleanup by granulocytes. **(f)** Relationship of total fetal biomass within a uterus (cohort biomass) to number of fetuses inside (surviving litter size in uterus) in saline and anakinra treatment groups shows a downward shift in the uninhibited control group. Histogram below shows the number of observations within each treatment group, following a trend towards larger surviving litter size in the saline group. *Treatment group “Meloxicam-1hr” was injected on days 11.7, 12.7, and 13.7 instead of 11.5, 12.5, and 13.5.

We tested the effects of counteracting fetal IL-1A using anakinra, a modified recombinant IL1RN that antagonizes the IL-1R1 receptor, effectively amplifying maternal inhibitory pathways. Pregnant females received daily subcutaneous injections on days 11.5, 12.5, and 13.5 postcopulation with either saline or anakinra. Tissue was collected six hours following the last injection at 13.75 dpc, and litter size and total fetal biomass were recorded to estimate maternal nutrient allocation (Fig 3a). Anakinra-treated animals showed a significant (two-tailed Welch’s *p* = 2.1 × 10^-2^) increase in fetal dry mass of 14% (from 7.97 to 9.10 mg; 95% CI: + 4% to +24%) (Fig 3b). This increase was coupled with a significant (two-tailed Welch’s *p* = 3.2 × 10^-2^) decrease in average litter size by 3.7 (from 12.0 to 8.3, 95% CI: −50% to −8%) (Fig 3c). One possible determinant of fetal biomass is a passive scaling effect: if space within the uterus is limiting, the size and number of offspring could be governed by a trade-off, where smaller litters have larger fetuses and larger litters face reduced fetal size due to crowding. If so, IL-1A could conceivably affect survival alone, with growth effects merely a byproduct of reduced crowding. However, cohort biomass showed a linear increase with litter size in each uterus (Fig 3d), ruling out passive scaling as the sole driver. Embryo resorption is a plausible mechanism to explain effects of late-gestation signaling on litter size: indeed, traces of actively resorbed embryos were occasionally observed in both untreated and treated uteri. These took the form of 1–2 mm white masses in the shape of fetuses (Fig 3e) nearly entirely consisting of granulocytes (Fig 3f), consistent with descriptions of uterine resorption in rodents [29]. The number of embryos captured during active resorption (9 across all animals) was insufficient to statistically determine frequency under different treatment conditions. These findings suggest that IL-1A helps marginal fetuses survive at the cost of individual growth, possibly through effects that stave off resorption.

### IL-6 blockade increases fetal biomass acquisition

To test the functional effects of IL-6, we conducted injection experiments with the same design as above using tocilizumab, a monoclonal antibody that competitively binds to the IL-6 receptor. Tocilizumab-treated animals showed a significant (two-tailed Welch’s *p* = 3.1 × 10^−2^) increase in fetal dry mass per fetus of 12% (from 7.97 to 8.90 mg; 95% CI: +4% to +21%) (Fig 3b), but no significant change in average litter size (12.0 versus 13.2, *p* = 0.372) (Fig 3c). This caused a suggestive, but non-significant trend in the opposite direction towards greater total maternal investment (+20 mg of average total litter biomass, *p* = 0.127) with IL-6 suppression. These results suggested that IL-6 signaling is, like IL-1A, associated with a small negative effect on fetal growth, but unlike IL-1A, does not significantly affect survival.

### PGE_2_ inhibition does not affect fetal allocation

Syncytial knots also express *PTGES* (Fig 2b and 2c), and the uterine concentration of this enzyme’s product, PGE_2_, increases approximately 20-fold in opossum late gestation [27]. We attempted to block this increase using daily injections of meloxicam during the last 3 days of gestation. Meloxicam is a specific inhibitor of the prostaglandin H_2_ synthase PTGS2, which produces the substrate that *PTGES* turns into PGE_2_. However, these injections failed to reduce uterine PGE_2_ levels as measured 6 hours following the last dose (45 ng/mg protein versus 55 ng/mg, *p* = 0.66) (S1 Fig), or to alter fetal biomass. An injection protocol delaying each injection by 5 hrs, with collection 1 hr following the last dose, reduced PGE_2_–9 ng/mg protein (*p* = 1.1 × 10^−6^) and flattened the observed association between uterine PGE_2_ levels and litter size (S1 Fig), demonstrating that meloxicam is indeed effective in the opossum but its effects on PGE_2_ are short-lived, likely due to rapid drug metabolism. This short-term decrease in PGE_2_ levels still showed no effect on biomass or litter size (Fig 3b and 3c). Continuous delivery via osmotic pump (from 12.75 to 13.75 dpc) lowered PGE_2_ to a similar degree (11 ng/mg), but showed no effect on biomass or litter size after 3 trials (S1 Fig) and thus trials were ceased. We conclude that depletion of prostaglandins is insufficient to alter nutrient allocation.

## Discussion

Our findings suggest that inflammatory signals in *M. domestica* play complex roles in shaping maternal investment. Cytokines such as IL-1A, IL-6, IL-10, IL-17A, and PGE2 are traditionally associated with immune activation, yet at the opossum fetal-maternal interface, their receptors are primarily expressed on non-immune maternal targets such as glandular epithelium, vascular endothelium, and fibroblasts. Functional experiments on two of these pathways, IL-1 and IL-6, demonstrate that neither has the properties expected of simple solicitation signals positively associated with fetal growth; inhibition of both did not reduce fetal biomass, but rather increased it. These signals appear to act beyond direct nutrient and immune regulation to affect fetal survival.

Once the shell coat degrades at 12 dpc, fetuses secure placental territory and receive oxygen and nutrients from endometrial vasculature and glands. One day later, the final, syncytial stage of placentation begins with the differentiation of giant syncytial knot cells. A strong candidate function for syncytial knot cells is to exert paracrine influences on endometrial development, evidenced not just by their prodigious production of cytokines (Fig 2a), but also by their production of the potent and highly diffusible truncated *VEGFA* isoform (VEGF_111_), a variant previously identified in the placenta of another marsupial, the dunnart [30]. *VEGF*_*111*_ expression in rodent models has shown that it promotes development of vascular networks [26]. Indeed, we find that placental cytokine expression temporally correlates with endothelial gene expression changes indicating the loss of tight junctions and altered vascular tone (Fig 2e). This lends transcriptomic evidence to previous histological work which revealed that the short placentation phase of *Monodelphis* pregnancy involves development of an endometrial capillary network that is absent in pseudopregnant animals [18], suggesting that its development requires fetal presence. These findings converge on a scenario where placental cells have evolved to actively remodel the endometrium to enable continued fetal development.

Our original hypothesis, that placental cytokines benefit fetuses by inducing inflammatory sequelae that enhance placental nutrient transfer, was not supported. Surprisingly, pharmacological inhibition of IL-1 and IL-6 did not reduce fetal biomass, but rather increased it, implying that both are slightly deleterious to fetal growth. Neither behaves as a simple solicitation signal that positively correlates with growth.

Why do opossum fetuses produce signals that apparently restrict their own growth? Our findings suggest that IL-1A helps marginal fetuses survive. Siblings may vie for capillary access in a manner resembling offspring begging in other species [31]. Not all are successful; fetuses were observed in the process of resorption in both saline and inhibitor-treated pregnancies (Fig 3d and 3e). Indeed, intrauterine resorption is routine in *M. domestica*: reports comparing surviving litter sizes to corpora lutea counts (a proxy for ovulation rate) suggest an average of 2−3 resorptions per pregnancy [19]. Abundant production of IL-1 sequestration agents (*IL1R2*) and receptor inhibitors (*IL1RN*) by maternal cells suggests that the mother actively suppresses IL-1 receptor activation in her own tissues. This could be interpreted as a form of maternal resistance to fetal solicitation: fetuses benefit from signaling that rescues marginal siblings, while the mother curbs excessively large litters by establishing an inhibitory bar that fetal signaling must surpass, as well as a tax on survival solicitation in the form of reduced growth (S2 Fig). Whether this pattern results from sibling competition, parental conflict with (a cooperating cohort of) offspring, or another evolutionary dynamic will require further investigation.

The function of placental IL-6 was less clear, as it appears to restrict growth without a benefit to survival. One possibility is that a compensating benefit to placental IL-6 in late gestation is realized only after our measurement window, for instance by influencing the timing of parturition or onset of lactation. Circulating IL-6 has metabolic effects, driving energy mobilization via lipolysis of fat stores [32]. Furthermore, in eutherian mammals with epitheliochorial placentas such as pigs, IL-6 is inferred to be anti-inflammatory and aid development of placental villi [33]. IL-6 is pro-inflammatory via receptor-independent signaling, but has anti-inflammatory effects associated with signaling through IL-6R [34]. The latter is widely expressed in opossum uterine cells (Fig 2c). Alternatively, if IL-6 once was a nutrient solicitation signal, it is possible that the mother has evolutionarily modified or dampened the endometrial response to IL-6, one of the co-evolutionary outcomes that can arise from evolutionary modification to generic signaling pathways when co-opted for communication.

Offspring solicitation signals have been extensively modeled under honest signaling models that predict that the only way for solicitation signals to be evolutionarily stable is if they carry associated costs [31,35–37]. When faced with a similar discovery in birds, Grafen [35] asked that the idea “that nestlings beg so noisily because it reduces their growth… not be rejected on the grounds that [it is] simply absurd”. Some aspects of solicitation theory, developed mostly in the context of parent-offspring conflict over allocation, carry over to marsupial pregnancy whereas others may be misleading. First of all, our experiments inhibited receptors rather than ligand production itself. As such, the observed effects must have been mediated by the physiological consequences of fetal cytokines on the mother, rather than by the metabolic cost to the fetus of producing signals, which is usually assumed in models of costly begging. More importantly, a second difference has to do with the magnitude of resources at stake. Marsupials invest comparatively little into offspring during placentation compared to lactation, making up an approximately 50:1 ratio in *M. domestica* [4,38–40]. The observed 12%–14% differences in allocation during placentation after IL-1 or IL-6 inhibition likely have little energetic cost to the mother, whereas each additional surviving offspring imposes a much greater energy transfer cost on mothers during lactation (around 1/10 of her biomass). Thus, survival is likely a more important determinant of maternal investment in marsupials than placental nutrient transfer. From this logic and our findings, we conclude that it is likely that fetal solicitation signals primarily target survival, whereas effects on growth are secondary.

Our results suggest that the immunological constraint hypothesis for marsupial gestation is no longer tenable. If inflammation were a damage-induced or alloantigen-triggered maternal reaction, one would expect pro-inflammatory signals to be expressed by maternal cells, i.e., as an inflammatory response to the nidatory injury. In marsupials (or at least in *Monodelphis*), however, these signals originate from fetal cells, and act mainly on non-immune targets. Physiological consequences of these cytokines also appear to differ: the direction of effect contrasts with known effects of inflammatory signaling in eutherian pregnancy, where exogenous IL-1A administration in mice is demonstrated to increase fetal death through inflammatory resorption [41]. The possibility that, in early viviparous mammals, maternal inflammation in response to fetal proteases was an ancestral trait that was secondarily modified has been discussed previously [9,12].

The present study has several limitations. First, it was not possible to measure both biomass and gestation length at the same time, as *M. domestica* females frequently consume some of their neonates. Placental cytokine signaling could advance parturition, as IL-1A, IL-6, and IL-17A are shown to induce premature delivery in humans and mice [42–45], and *IL1R2*-activating polymorphisms reduce the risk of preterm birth in humans [46]. Future investigation into effects of placental inflammatory signaling on gestation length is warranted. Second, our vascular-remodeling interpretation remains indirect, and direct mechanistic experiments on the density and permeability of the capillary bed will be required to substantiate this interpretation. Third, our study design did not allow inter-individual variation in biomass or cytokine production among fetuses in the same uterus to be determined, which will be required to rigorously test for sibling competition. Sibling competition at the transition from placental to lactational provisioning has been proposed for other marsupial species that deliver more offspring than they have nipples, like the Virginia opossum and Tasmanian devil [47,48]. *M. domestica* females have 11−13 nipples [49] and a mean surviving litter size of around 9 at delivery [19], suggesting that intrauterine brood reduction plays a greater role in differential offspring survival than nipple access. If IL-1A promotes redistribution of resources to save marginal siblings, we predict that biomass variance should be increased when IL-1R is inhibited. Despite these limitations, our experiments allowed a cost of IL-1A and IL-6 signaling, and a positive effect of IL-1A on surviving litter size, to be observed.

Recently, we found that *IL1A* and *IL6* production during the short postattachment placentation stage of pregnancy is shared between *Monodelphis* and the fat-tailed dunnart *Smithnopsis crassicaudata,* whereas the tammar wallaby *Macropus eugenii* expresses *IL6* but not *IL1A* at appreciable levels [8]. The dunnart has an average litter size of 7, similar to *M. domestica*, whereas the wallaby has singleton births [38]. If litter size is regulated by gestational IL-1A in marsupials as our empirical results suggest, this phylogenetic distribution is consistent with a lack of functional utility for fetal IL-1A in a singleton species. Outside of mammals, the viviparous skink *Chalcides chalcides* has a yolk sac placenta analogous to the opossum, a mean litter size of 7.8 [50], and similarly shows expression of IL-1A in its placenta [51]. Further investigation of the role of placental cytokines in vertebrates with diverse reproductive strategies is warranted. While our model of uterine capillary development as the factor determining differential offspring survival remains speculative, it presents testable predictions for future investigation into a surprisingly rich and unusual form of fetal–maternal relationship.

### Broader evolutionary context

We can place the opossum’s fetal-maternal signaling into the trajectory of mammalian viviparity more broadly. The common ancestor of mammals is inferred to have reproduced by matrotrophic oviparity, the state retained in monotremes where embryos are nourished by uterine secretions across the proteinaceous shell coat [52]. From this state, precocious production of hatching proteases, leading to a short intrauterine period with direct fetal-maternal contact. This period carried substantial benefits because of the ready availability of maternal nutrients unencumbered by egg covers and in opossum still is associated with rapid fetal development. Continued protease secretion after hatching—conserved across opossums, humans, and rodents [23]—irritates the uterine luminal epithelium [53]. Mucosal inflammation leads to angiogenesis and vascular leakage at the site of fetal attachment, as well as neutrophil and monocyte recruitment. It can be hypothesized that initially, the former was beneficial to fetal development while the latter was detrimental. This provided raw material for natural selection to prune harmful signaling interactions while elaborating useful ones - a process likely central to the evolution of vertebrate placentas [54,55].

Our results suggest that the present state in marsupials is not just the result of reductive pruning, nor is it a crude unpruned state as early constraint theories alleged [1,2], but it is also the product of lineage-specific innovation. Thus, our findings are consistent with the view that marsupial reproduction is derived rather than primitive [5]. Previous iterations of this argument have focused on the elaboration of lactation as an alternative strategy to eutherian-like placental provisioning [3,4], but here we find that the marsupial-eutherian dichotomy extends to the use of inflammatory signals in gestation as well. Evidence for pruning include maternal expression of IL-1 antagonists *IL1RN* and *IL1R2*, and the fact that uterine leukocytes largely lack *IL1R1* expression, preventing positive feedback amplifying inflammatory signaling. Angiogenic fetal signals such as *IL1A* and a truncated isoform of *VEGFA* appear to have acquired developmental functions beyond just byproducts of an immune reaction, and it is possibly through these effects that IL-1A exerts a positive effect on fetal survival. From that stage onwards, the marsupial and eutherian lineages took divergent evolutionary paths, and in both, the fetal-maternal interaction was extensively modified. Whereas eutherians evolved mechanisms to suppress certain inflammatory pathways such as IL-17 [10], and co-opted others to support an implantation that allows extended gestation [6], marsupials maintain a short period of direct fetal-maternal contact, and their fetuses appear to have co-opted inflammatory mediators to secure development of endometrial blood supply while mothers limit the response of the maternal immune cells by suppressing IL-1 receptors. We propose that fetal-maternal communication in the opossum strikes an evolutionarily negotiated balance between maximal fetal survival, brood reduction by the mother to prevent over-investment, and the risk of runaway inflammatory reaction.

### Clinical implications

Meloxicam is regularly administered as a veterinary analgesic to gray short-tailed opossums at low doses of 0.2 mg/kg [56]. Our failure to demonstrate an effect of meloxicam on local PGE_2_ levels 6 hours post-injection with a dose of 2 mg/kg (S1 Fig) suggests that it is rapidly metabolized. The metabolic rate of meloxicam has been shown to be elevated in several other marsupials (brushtail possums, koalas, and ringtail possums) compared to eutherian species like rats and dogs by an order of magnitude, with a half-life on the order of hours [57]. The efficacy of meloxicam in marsupials may therefore be overestimated and treatment regimes may be in need of reconsideration. More encouragingly, our observed biomass effect of tocilizumab suggests that it is functionally active in *M. domestica*, unlike the mouse [58]. Despite their phylogenetic distance, the opossum exhibits greater IL6RA sequence conservation with the human than does the mouse, including at the 6 amino acid positions known to bind tocilizumab [58]: 4 of these residues are identical, while the other 2 are substituted with amino acids with similar charge properties (R → Q and E → K) (S3 Fig). The opossum may therefore be a more suitable model organism for tocilizumab than murine counterparts.

## Materials and methods

### Animal husbandry

*M. domestica* were raised in a breeding colony at Yale University according to established technical protocols [59]. Male and female animals were housed separately after 3 months of age, at which point female individuals were introduced to the male room for sexual preconditioning. Breeding was attempted after 6 months of age. Non-cycling female opossums were introduced to the male room for 1 day, and subsequently swapped into the used cage of a prospective male partner for 5 days. Afterwards, both individuals were placed into a breeding cage and video recorded to assess the time of copulation. If multiple copulations were observed, the first was used to calibrate 0 dpc.

### Injection trials

Female opossums were mated as outlined in our husbandry protocol. On day 11.5 of pregnancy, females were assigned to treatment groups of anti-inflammatory (meloxicam, anakinra, or tocilizumab) or a vehicular control of saline. Meloxicam was administered at a dose of 0.2 mg/animal (2 mg/kg for an average 100 g female). Anakinra was administered at a dose of 10 mg/animal following manufacturer’s suggestion based on preclinical studies. Tocilizumab was administered at a dose of 2.5 mg/animal based on the allometric scaling approach of Nair and Jacob [60], by which this dosage was determined as the equivalent to a 4 mg/kg human dose.

Unless otherwise noted, injections were given on day 11.5, 12.5, and 13.5, and animals were necropsied 6 hours following the last injection (at day 13.75). The ovarian-facing top-third of each uterus was flash-frozen in liquid nitrogen and stored at −80 °C for small molecule extraction, and fetuses were collected into pre-dessicated glass vials divided by uterus of origin (two from each animal) for dry weight. Fetuses were desiccated in a 60 °C oven for at least 72 hours before weighing.

### Osmotic pump implantation

Female opossums were again mated as outlined in our husbandry protocol. On day 12.75 of pregnancy, females were placed in one of two conditions, meloxicam treatment or a vehicular control of saline. Meloxicam and vehicular control were administered via an Alzet osmopump 2001D according to the manufacturer’s recommendations. Pumps were loaded with 200 µL of veterinary grade meloxicam (Boehringer Ingelheim Metacam, 5 mg/mL injectable product), equaling a total of 1 mg to match the syringe injections. Given the properties of the pump, meloxicam was released at a constant rate of ~ 8 μL per hour over a 24-hour period.

Pumps were implanted subcutaneously on the dorsal surface posterior to the scapulae. Animals were anesthetized using controlled isoflurane administration and held in sternal recumbency, and the implantation site was shaved with clippers and washed with povidone-iodine. Pumps were inserted via a mid-scapular incision and the wound closed with wound clips. At 13.75 days from copulation, 24 hours following osmopump implantation, opossums were necropsied and tissue and fetuses were collected as above.

### Quantification of prostaglandin E_*2*_

Flash-frozen uterine samples were sent to Cayman Contract Services (Ann Arbor, MI) for commercial quantification of prostaglandin E_2_ using enzyme-linked immunosorbent assay (ELISA) (Cayman 514010 kit; monoclonal antibody #414013). Protein was quantified by bicinchoninic acid assay (BCA; Cayman 701780 kit) and used to normalize PGE_2_ concentrations.

### Statistical analysis

Sample sizes were determined by availability of animals. A total of 42 animals were used, consisting of *n* = 8 injected with saline, *n* = 14 injected with meloxicam using standard injection timing, *n* = 6 injected with meloxicam using a 5-hour shifted dosing, *n* = 7 injected with anakinra, *n* = 5 injected with tocilizumab, *n* = 3 implanted with meloxicam-containing osmotic pumps, and *n* = 2 implanted with saline-containing osmotic pumps. Two additional experiments (1 saline, 1 tocilizumab) lacked biomass data due to damage of embryos during sample collection. Individuals that were not pregnant upon dissection, showing no signs of ovulation or uterine cycling, were excluded from the study and samples were not collected.

Opossums have two fully separate uteri, and offspring in both are almost always fertilized, and delivered, simultaneously. Given this peculiarity, per-uterus measurements were first obtained and then aggregated to yield per-animal metrics (sum for litter sizes, mean for biomass per fetus across both uteri). For transparency, statistical analyses for which per-uterus data were available were conducted both using aggregated per-animal metrics, and using a nested statistical model to directly compare per-uterus measurements as outlined below. Key findings did not substantially differ between approaches.

To evaluate treatment effects on biomass per fetus and litter size while accounting for non-independence of left and right uteri from the same animal, we fit linear mixed-effects models using statsmodels mixedlm (v0.14.2) with the formulas “BiomassPerFetus ~ C(Treatment)” and “LitterSize ~ C(Treatment)”, where Treatment was modeled as a categorical fixed effect and a random intercept was included for each animal. Models were fit by restricted maximum likelihood, Wald tests from the fitted model were used to obtain *p*-values for fixed effects, which are reported as “Nested p” in Fig 3. In addition, treatment effects on biomass per fetus and litter size (aggregated between both uteri within each animal) were tested using two-tailed Welch’s t-tests via the stats.ttest_ind() function in the scipy package (v1.15.2), the results of which are reported as “Welch’s p” in Fig 3. Results from both approaches are reported in order to assess robustness of statistical findings. Treatment effect sizes were quantified as the percent change in arithmetic mean biomass per fetus relative to saline, and 95% confidence intervals were estimated by nonparametric bootstrap using scipy.stats.bootstrap() function with the method parameter set to “percentile” and number of resamples set to 10,000.

As anakinra treatment showed significant effects on both biomass and surviving litter size, analysis of covariance was used to assess whether the treatment effect on these two variables was statistically independent. All per-uterus measurements of fetal biomass and surviving litter size from saline-treated and anakinra-treated individuals where both measurements were available were used for this analysis. The model formula (“Biomass ~ Litter Size * Treatment”) treated total fetal biomass as the dependent variable, surviving litter size as a covariate, treatment group as a categorical predictor, and contained an interaction term. The model was fit using ordinary least squares via the ols() function and statistical significance was assessed using Type II sums of squares using the anova_lm() function, both in the statsmodels package as above.

### Laser microdissection and micro-bulk RNA sequencing

Placental-only transcriptomes were captured from laser microdissected samples of fetal tissues directly apposing the uterine lumen in cryosections of 13.5 dpc pregnant uteri (biological *n* = 3). Uteri to be used for this procedure were dissected into phosphate-buffered saline and subsequently immersed Tissue-Tek OCT compound (Sakura Finetek, 4583) in a block mold and flash frozen by exposure to a bath of isopentane surrounded by dry ice, and stored at −80 °C. 14-μm sections were prepared from the resulting blocks on a cryomicrotome (Microm HM 500 OM) and placed onto polyethylene naphthalate membrane slides (Leica Microsystems, 11505158) sterilized by ultraviolet irradiation in a biosafety cabinet (Baker SterilGARD, SG-404) for 30 min. Sections were processed on a Leica LMD7000 apparatus and excised tissue dropped directly into lysis buffer for processing using a Qiagen RNeasy Micro Kit (74004). RNA libraries were prepared by the Yale Center for Genome Analysis using a NEBNext Low Input Library Prep Kit (E6420) and sequenced using an Illumina NovaSeq apparatus.

### Transcriptomic analysis

Single-cell transcriptomes from the fetal-maternal interface of untreated pregnant *M. domestica* at 13.5 dpc and non-pregnant animals were obtained from recently published studies [11,23] (NCBI Gene Expression Omnibus GSE274701 and GSE292958). Differential expression analysis between vascular endothelial cells in 13.5 dpc and non-pregnant animals was conducted using likelihood ratio testing in MAST v3.20 [61]. For MAST, pregnancy status and biological replicate were modeled as fixed effects in a generalized linear model (method = “bayesglm”), with the number of genes expressed per cell included as a covariate.

Cell-cell interactions were inferred from single-cell data using simple expression thresholding (chinpy v0.0.57, https://gitlab.com/dnjst/chinpy) complemented by statistical analysis of cell type specificity using the LIANA+ (v1.5.1) [62] rank_aggregate() function. A custom ligand-receptor ground truth database was adapted from CellPhoneDB v5.0.0 [63] to only include inflammatory cytokines with 1:1 orthologs between human and opossum (archived at https://gitlab.com/dnjst/antiphlogistic_opossum). Only interactions passing expression thresholds 40 counts per million and 15% of cells in the sending and receiving cell populations were retained.

For all bulk RNA sequencing data, raw reads were aligned to the *M. domestica* genome (Ensembl version 104) and quantified using kallisto v0.45.0 [64]. Statistical testing for differential expression in bulk data was conducted using the DESeq2 method as implemented in pyDESeq2 v0.5.0 [65]. *P*-values reported for differential expression between conditions in Fig 2 are Wald test values are pyDESeq2 Benjamini-Hochberg-adjusted.

### Ethics statement

All experiments and animal care were conducted following ethical protocols approved by the Yale University Institutional Animal Care and Use Committee (2015-11313 and 2020-11313). Animal care and use adhered to the U.S. Animal Welfare Act and USDA Animal Welfare Regulations. Euthanasia was conducted in accordance with the AVMA Guidelines for the Euthanasia of Animals.

## Supporting information

S1 Fig Prostaglandin production under anti-inflammatory treatment. **(a)** Uterine concentrations of PGE_2_ (ng/mg protein) after treatment with meloxicam. The middle lines mark the median, boxes mark the interquartile range (IQR), and whiskers extend to the furthest points not exceeding 1.5× the IQR. **(b)** Regression plot of PGE_2_ concentrations versus number of embryos in the uterus shows a positive association in the absence of inhibition. Coefficients and one-sided Pearson correlation test *p*-values (H₁: *ρ* > 0) were calculated using the scipy.stats.pearsonr function. **(c)** Biomass per fetus remained unchanged after 24 hours of constant infusion via implanted osmopump during days 12.75–13.75 of gestation. Full measurements are in S5 Data.
(TIF)

S2 Fig Conceptual model of IL1A’s opposing effects on survival and growth. In this model, intrauterine resorption from an initial cohort of fertilized zygotes is commonplace and influenced by the balance between expression of fetal IL-1A promoting survival and maternal IL1RN and IL1R2 promoting resorption. In normal pregnancies, this balance leads to a low level of resorption and litter size before birth of around 12 (right side). Interventions tipping the balance towards greater inhibition (i.e., by supplemental IL1RN, anakinra), increases resorption (left side). Embryo illustrations are drawn after [66].
(TIF)

S3 Fig Amino acid alignment between human and opossum IL-6 receptors shows greater similarity to each other than either does with mouse. **(a)** Human-opossum alignment of the translated IL6RA peptide. Amino acids constituting the binding site in the human peptide are marked in purple. Substitution codes: * = identical,: = same chemical properties,. = weak similarity, - = insertion/deletion. **(b)** NCBI BLAST dendrogram showing greater similarity of opossum and human peptides to each other than to mouse Il6ra, despite the shorter phylogenetic distance between human and mouse.
(TIF)

S1 Data Bulk RNA expression (TPM) and differential gene expression testing results between 13.5 dpc placenta, 12.5 dpc placenta [21] and 13.5 dpc-equivalent pseudopregnant uterus [18]. Wald test statistics are output from pairwise pyDESeq2 [65] tests between 13.5 dpc placenta and the two indicated comparison sets. Counts from kallisto pseudoalignment [64] were used for statistical analysis, while normalized expression values are also reported and used for plotting.
(XLSX)

S2 Data Inferred inflammation-related ligand-receptor signaling at the 13.5 dpc fetal-maternal interface by LIANA+ [22]. Expression data and original ligand-receptor list were taken from [11], and subset to inflammatory pathways appearing in Fig 2’s bulk RNA-seq analysis. Specificity Rank and Magnitude Rank refer to LIANA+ rank aggregate test *p*-values for specificity and magnitude-related statistical significance. Receptors or ligands composed of multiple subunits are separated by an underscore, and the gene with the lower expression was used to calculate scores.
(XLSX)

S3 Data MAST differential expression statistics, expression values, and percent of cells expressing genes related to vascular permeability and remodeling in vascular endothelial cells (VEC). Data are obtained from single-cell transcriptomes of the non-pregnant (NP) and 13.5 dpc uterus.
(XLSX)

S4 Data Expression of *VEGFA* isoforms in 13.5 dpc placenta and uterus. Transcript-level counts are collated from kallisto pseudoalignment output [64] before gene collapsing was performed, and annotated to the corresponding protein in the Ensembl database. Percent counts of each isoform represent the percentage of all transcripts among all three replicates belonging to that isoform.
(XLSX)

S5 Data Biomass measurements and litter size recordings from anti-inflammatory inhibitor experiments and their controls. “White bodies” and “red bodies” represent numbers of resorbed fetal remains (see Fig 3) observed, and are not included in litter size calculations. Litter sizes reported are per-uterus, with “uterus side” listing left (L) and right (R) from the same animal ID. Biomass represents post-dehydration mass. PGE_2_ levels were measured by Cayman Contract Services in both absolute levels (pg/mL) and relative to protein mass in the same tissue (ng/mg protein).
(XLSX)

## Abbreviations

dpc: days postcopulation
IL-1A: interleukin-1A
IL-6: interleukin-6
TPM: transcripts per million
