# Supplementary figures and images for: Fetal inflammatory signals regulate maternal investment during marsupial pregnancy
Source: PLoS Biol. 2026 Feb 24;24(2):e3003670. doi: 10.1371/journal.pbio.3003670 (PMC12981560; doi:10.1371/journal.pbio.3003670)

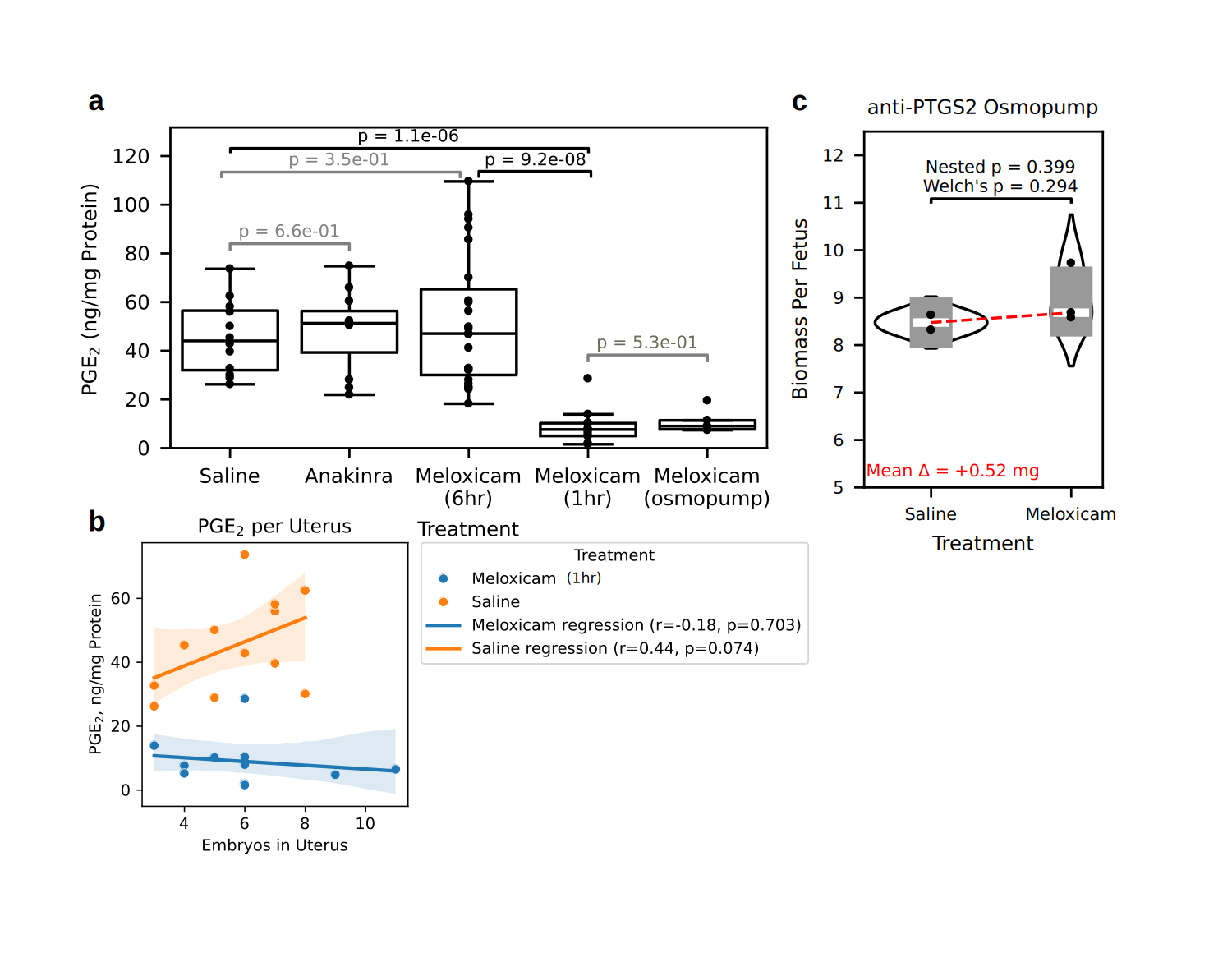

Supplement: S1 Fig — (a) Uterine concentrations of PGE2 (ng/mg protein) after treatment with meloxicam. The middle lines mark the median, boxes mark the interquartile range (IQR), and whiskers extend to the furthest points not exceeding 1.5× the IQR. (b) Regression plot of PGE2 concentrations versus number of embryos in the uterus shows a positive association in the absence of inhibition. Coefficients and one-sided Pearson correlation test p-values (H₁: ρ > 0) were calculated using the scipy.stats.pearsonr function. (c) Biomass per fetus remained unchanged after 24 hours of constant infusion via implanted osmopump during days 12.75–13.75 of gestation. Full measurements are in S5 Data. (TIF) [file pbio.3003670.s001.tif]

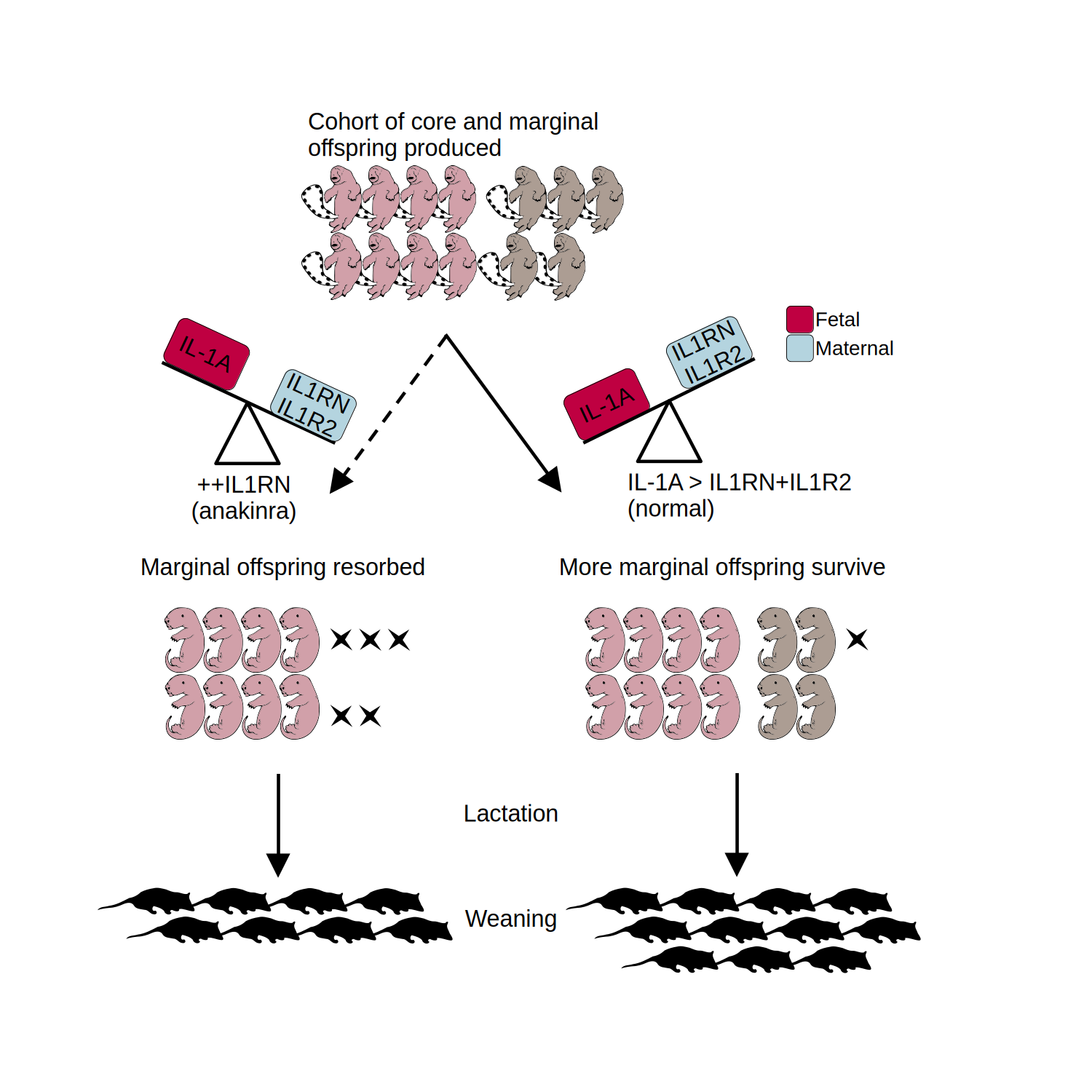

Supplement: S2 Fig — In this model, intrauterine resorption from an initial cohort of fertilized zygotes is commonplace and influenced by the balance between expression of fetal IL-1A promoting survival and maternal IL1RN and IL1R2 promoting resorption. In normal pregnancies, this balance leads to a low level of resorption and litter size before birth of around 12 (right side). Interventions tipping the balance towards greater inhibition (i.e., by supplemental IL1RN, anakinra), increases resorption (left side). Embryo illustrations are drawn after [66]. (TIF) [file pbio.3003670.s002.tif]

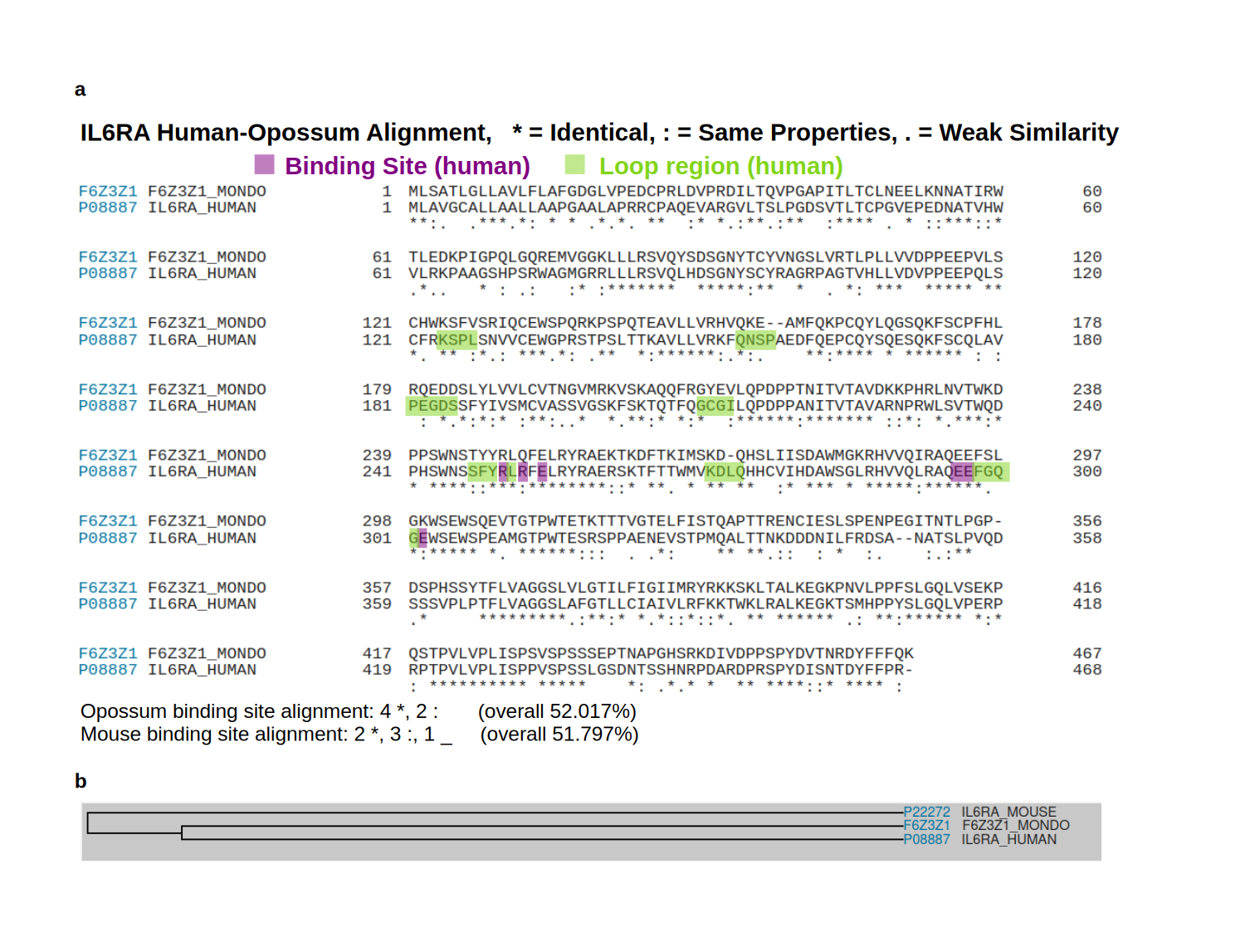

Supplement: S3 Fig — (a) Human-opossum alignment of the translated IL6RA peptide. Amino acids constituting the binding site in the human peptide are marked in purple. Substitution codes: * = identical,: = same chemical properties,. = weak similarity, - = insertion/deletion. (b) NCBI BLAST dendrogram showing greater similarity of opossum and human peptides to each other than to mouse Il6ra, despite the shorter phylogenetic distance between human and mouse. (TIF) [file pbio.3003670.s003.tif]
